# Features of Heat Treatment the Ti-6Al-4V GTD Blades Manufactured by DLD Additive Technology

**DOI:** 10.3390/ma14154159

**Published:** 2021-07-27

**Authors:** Marina Gushchina, Gleb Turichin, Olga Klimova-Korsmik, Konstantin Babkin, Lyubov Maggeramova

**Affiliations:** 1 World-Class Research Center “Advanced Digital Technologies”, State Marine Technical University, 190121 Saint-Petersburg, Russia; gleb@ltc.ru (G.T.); o.klimova@ltc.ru (O.K.-K.); babkin@ilwt.smtu.ru (K.B.); 2 Central Institute of Aviation Motors (CIAM), 111116 Moscow, Russia; mag@ciam.ru

**Keywords:** Ti-6Al-4V, direct energy deposition, thermal history, annealing, phase composition, microstructure, tensile properties

## Abstract

Additive manufacturing of titanium alloys is one of the fastest growing areas of 3D metal printing. The use of AM methods for parts production in the aviation industry is especially promising. During the deposition of products with differently sized cross-sections, the thermal history changes, which leads to non-uniformity of the structure and properties. Such heterogeneity can lead to failure of the product during operation. The structure of deposited parts, depending on the thermal cycle, may consist of α’, α + α’ + β’, and α + β in different ratios. This problem can be solved by using heat treatment (HT). This paper presents research aimed towards the determination of optimal heat treatment parameters that allows the reception of the uniform formation of properties in the after-treatment state, regardless of the initial structure and properties, using the example of a deposited Ti-6Al-4V gas turbine blade.

## 1. Introduction

The transition of the world aircraft industry to innovative technologies, including the replacement of metal structures with composite materials, the development of additive manufacturing, and the introduction of new artificial intelligence systems in the aircraft control system, is becoming an increasingly relevant trend.

Computer engineering is widely used to create new materials in the aviation industry, which reduces the cost of the production of expensive full-scale prototypes by using virtual models. Additive technologies, most commonly based on the use of virtual models, are widely available due to the fact that they allow the manufacturing of products with complex geometric shapes and profiles. The use of such advanced technologies will significantly reduce the time introduction of products to the market and their cost, reduce material consumption, and reduce the product failure, which is a clear advantage for use in most industries [1,2,3].

The aviation industry is characterized by increased requirements for structural materials, and in some cases is a main customer and consumer of new materials and technologies [4]. A range of AM techniques are now available. The following additive manufacturing methods have become particularly popular in the aircraft industry for metal parts: selective laser melting (SLM), electron beam melting (EBM), and direct laser deposition (DLD). In SLM and EBM, thin powder layers are consolidated layer by layer using electron or laser beam scanning, and a layer is formed along the path of the corresponding user-created model [5,6]. In DLD, the formation of a layer occurs by the coaxial supply of laser radiation and powder through special nozzles. Each of the methods has its advantages and disadvantages [7]. Due to the design features of the machines, SLM and EBM are more suitable for small- and medium-sized products, while DLD is attractive due to the possibility of producing large-sized products [7,8].

DLD is applicable to producing products from alloys based on iron, titanium, and nickel, as well as composite materials or compositionally graded materials. The α + β titanium alloy Ti-6Al-4V is widely used in aerospace applications, and much research has been conducted on AM with this alloy. There are many works devoted to the study of the structure and properties of additively manufactured Ti-6Al-4V in the building as well as the post-processing state [9,10,11,12]. Heat treatments (HTs) are solved problems of structural and phase inhomogeneity, residual stresses, and anisotropy. Hot isostatic pressing (HIP) of deposited samples allows the removal of defects, such as pores and non-fusion [13]. 

The fundamental possibility of achieving high strength and fatigue properties of model samples deposited from Ti-6Al-4V powder is shown in a number of works [14,15,16,17,18]. However, in most studies, the represented data for model samples have the form of plates, which are far from real parts. For example, [19,20] presented an investigation regarding the influence of product shape and thickness on other properties, and it was found that the shape has a more significant effect on thin-walled samples than on thick-walled samples. The overall dimensions of the samples also affected the properties: samples with a thick wall had higher strength, which, according to the authors, is associated with the size and morphology of the initial β-grain [21,22]. Thus, we can assume that the combination of thin-walled and thick-walled elements in one part can lead to significant heterogeneity of properties and structures throughout the product, which can negatively affect the performance of the entire part.

In this particular case, this problem can be solved by careful and accurate selection of modes, which requires a significant amount of work. On a global scale, the problem can be solved by developing the optimal heat treatment parameters that, regardless of the initial phase composition and grain size, the uniform structure and properties can provide.

This paper presents studies on the developing and selection of optimal heat treatment modes that ensure the uniform formation of properties in the deposited compressor gas turbine engine (GTE) blades of a Ti-6Al-4V after heat treatment, regardless of the initial structure and properties. In this work, we investigated a wide range of HT temperatures for laser deposited Ti-6Al-4V to establish the relationships between heating temperature and the mechanical properties. The selected mode was tested for samples with different initial microstructures and phase compositions.

## 2. Materials and Methods

**Figure 1:**
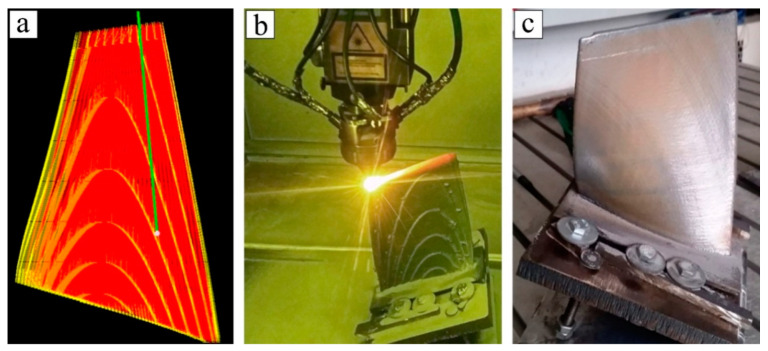
The blades of a gas turbine compressor; (**a**) the path generated in Additive Control; (**b**) the process of blade deposition; (**c**) for a Ti-Al-4V deposited blade.

All the compressor gas turbine engine blades investigated in this research were built using the Ti-6Al-4V titanium alloy powder, with a fraction of 45–90 microns produced by PREP (plasma rotating electrode process). Samples were produced by the robotic complex developed at St. Petersburg State Marine University. The complex includes an LS-5 fiber laser, an anthropomorphic robot, a 6 m^3^ protecting chamber, a two-axis positioner, a powder feeder, and a control system. The path was generated in the Additive Control 1.0 program (ILWT, Saint Petersburg, Russia) using a 3D model of the blade. Figure 1a shows the trajectory of the deposition of the blade. The technological parameters for a Ti-6Al-4V alloy are presented in Table 1. The process of blade deposition and the final result are shown in Figure 1b,c. The process was carried out in a chamber with an argon atmosphere, and the oxygen level in the chamber was 2000 ppm.

The effect of heat treatment on the structural change was studied on the 5 × 5 × 10 mm^3^ pieces, which were cut from the central part of the deposited blades. The optimal heat treatment conditions for the Ti-6Al-4V titanium alloy were determined by varying the furnace heating temperature and holding time. The temperature varied within the range of 600–950 °C. The hold time was 2 h. 

**Figure 2:**
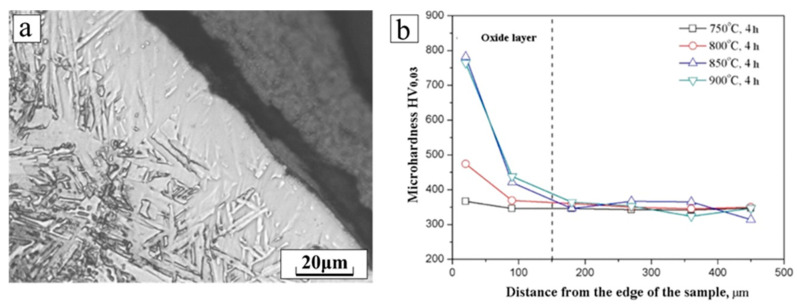
The formation of an oxide layer in deposited Ti-6Al-4V during heat treatment in an air atmosphere; (**a**) an oxide layer of Ti-6Al-4V after HT at 900 °C for 4 h; (**b**) the dependence of the oxide layer hardness of the holding temperature.

An air atmosphere was chosen for heat treatment in an SNOL furnace, without additional argon protection. This choice was made on the basis of preliminary studies conducted by the authors of this article. It was shown that at a temperature HT of 900 °C and a holding time of 4 h, the maximum thickness of the alpha case layer in Ti-6Al-4V, which consists of TiO_2_ and a Ti (O) solid solution, does not exceed 200 μm (Figure 2a).

At temperatures above 600 °C, titanium actively interacts with oxygen, and forms a solid solution of up to 10 at.% in α-Ti. The diffusion rate of oxygen atoms increases with increasing temperature and holding time, but the formation of TiO_2_ oxide on the surface decreases the diffusion rate. Thus, the effect of oxygen on the properties was insignificant when we used intervals of temperatures and time considered in the article for Ti-6Al-4V heat treatment. The assessment was based on hardness changes (Figure 2b).

Plates with a size of 75 × 15 × 35 mm^3^ for mechanical tests were deposited in accordance with the thermal cycles corresponding to blade deposition. For the definition of the mechanical characteristics of deposited products, uniaxial tensile tests were performed. Mechanical tests were performed on a universal testing machine, the Zwick/Roell Z250 Allround series (Zwick/Roell, Ulm-Einsingen, Germany). The standard cylindrical samples were cut from the printed parts according to geometry from the ASTM E8, with a gauge diameter of 6.0 mm and a gauge length of 24.0 mm. The sample displacement was recorded using an extensometer. The optical microscope DMI 5000 (Leica, Wetzlar, Germany) with the Axalit software (Axalit, Moscow, Russia) was used for microstructural analysis. The sample surfaces were polished with grit SiC papers up to 2500 grits, with further polishing by an aluminum oxide suspension of 1 μm and final polishing with colloidal silica. As a final step, the etching procedure was conducted. A solution of 93 mL of H2O + 2 mL of HF + 5 mL of HNO3 was applied to the polished sample surfaces for 40 s.

A Bruker Advance D8 diffractometer (Bruker, Billerica, USA) with CuKα radiation (wavelength = 1.5418 Å) was used to perform the XRD analysis. The detector was a LynxEye linear position-sensitive detector (PSD) with a capture angle of 3.2 degrees 2θ. The cross-sectional surface of the samples was polished with a grit SiC paper of 2500 grits, and, after etching, measured in the 2θ range of 30°–60° with a step size of 0.05 and an incremental time of 0.02.

Microhardness measurements were performed with an FM-310 microhardness tester (Future Tech, Kawasaki, Japan). The sample surface for testing was polished with 500 and 2500 grit SiC abrasive papers. Microhardness was measured on the central part of the deposited blades.

## 3. Results and Discussion

The recrystallization temperature range for the Ti-6Al-4V alloy was 850–950 °C. Including this, temperatures of 850, 900, and 950 °C were investigated for heat treatment deposited samples. The pre-crystallization annealing modes were also used, since these temperatures may be sufficient to relieve stresses and metastable phase decomposition.

At temperatures above 1000 °C, recrystallization and significant growth of alpha plates occurs. This leads to a decrease in the mechanical properties for AM samples; therefore, heat treatment above 1000 °C was not considered in this work [23]. The effect of the cooling rate on the microstructure after heat treatment was also not considered, since, in previous works, it was found that the cooling rate during HT does not have a significant effect [24]. Due to the very fine martensite, the kinetics were completely different compared to treatment of equiaxed or heavily deformed microstructures. Consequently, the application of standard heat treatments shows that these treatments do not lead to the usual or expected results [24].

Before 700 °C, a layer structure was still observed, which is typical for as-deposited samples. Annealing for 2 h at a temperature of 700 °C led to the formation of new equiaxed grain near the boundaries between the layers (suggesting that, in these areas, the greatest deformation of the primary grain occurred). Thus, a shift in the temperature of the onset of the recrystallization process was observed.

**Figure 3:**
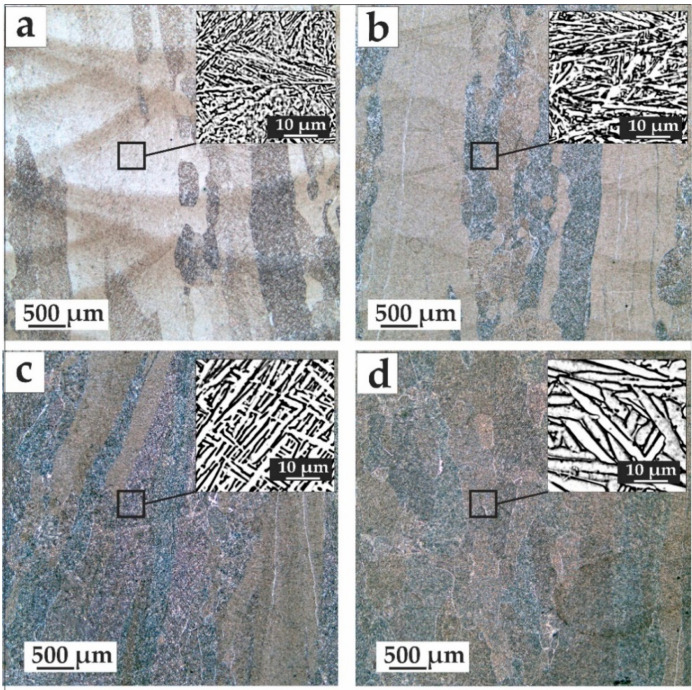
Microstructure of deposited Ti-6Al-4V samples after heat treatment (50× magnification): (**a**) 700 °C, (**b**) 850 °C, (**c**) 900 °C, 2 h, (**d**) 950 °C, 2 h.

This may be due to a high level of initial grain deformation due to high internal stresses (Figure 3a). Formation at the boundaries between the layers of secondary grains was also observed upon a heating temperature of 800 °C and a holding time of 2 h (Figure 3b). As the temperature rose to 900 °C, a more intense recrystallization process occurred, and the formation of new equiaxed grains occurred not only at the boundaries between the layers, but also inside the layer (Figure 3c,d). From the deformed grains, the growth of secondary under-formed grains was observed. With increasing holding time, the size of the secondary grains increased at 800–900 °C. Inside the grain, the morphology of the α-plates changed.

The temperature of 950 °C was the boundary of the end of the recrystallization process (for α + β titanium alloys produced by conventional methods [25]). At this temperature, a morphology of the α and β plates inside the grain changed (Figure 3a). In addition, due to the closer temperature of the allotropic transformation, partial recrystallization occurred due to the transition from the low-temperature α to the high-temperature β-phase, and vice versa, upon cooling in the air. The anisotropy of the alloy decreased because of the change in grain size.

In the process of heating to 600 and 650 °C and holding for 2 h, only residual stresses were removed, there were no visible structural changes, and the form of the α/α’ phase was the same, with a characteristic needle structure. The absence of the α’ decomposition was also evidenced by the high hardness of the samples similar to the initial state. Due to the diffusion process, increasing to a temperature to 700 °C led to the decomposition of the metastable phase, and a thickness increase of the α phase lamellae began (Figure 3a). The morphology also changed slightly; the needle structure was replaced by a lamellar structure. 

A heat treatment temperature of 800 °C with a holding time of 2 h led to the secondary α phase, which began to form along the grain boundaries, and the needles transformed into the plates (Figure 3b). There were both long and short plates located perpendicular to each other, as well as in separate packages of parallel plates.

During heat treatment with a temperature of 850 °C for 2 h, the secondary α phase was formed, and the metastable α’ phase was decomposed with the formation of stable composition a + β phases. The width plates increased, and the amount of secondary α increased as well (Figure 3c). At a temperature of 900 °C and a holding time of 2 h, the structural components grew. At an annealing temperature of 950 °C, partial recrystallization occurred due to allotropic transformation, and a structure of the “basket weaving” type was formed. The thickness of the plates increased significantly in comparison with the annealing temperature of 850 °C.

At low annealing temperatures (below 600 °C), the decomposition of a’ was incomplete, as is shown by the low value of the hardness.

### 3.1. Heat Treatment Temperature Effect on Mechanical Properties

Mechanical test results are presented in Table 2. It can be seen that temperature and time increasing led to YS reduction. On the contrary, an increase in elongation occurred up to a temperature of 900 °C; above this temperature, with a holding time of 2 h, a decrease in the relative elongation occurred. H. Galarraga et al. presented the influence of heat treatment by using different modes that consisted of several stages of electron beam-melted Ti-6Al-4V. However, for DLD, it is possible to obtain high mechanical properties using a one-stage heat treatment [12]. 

**Figure 4:**
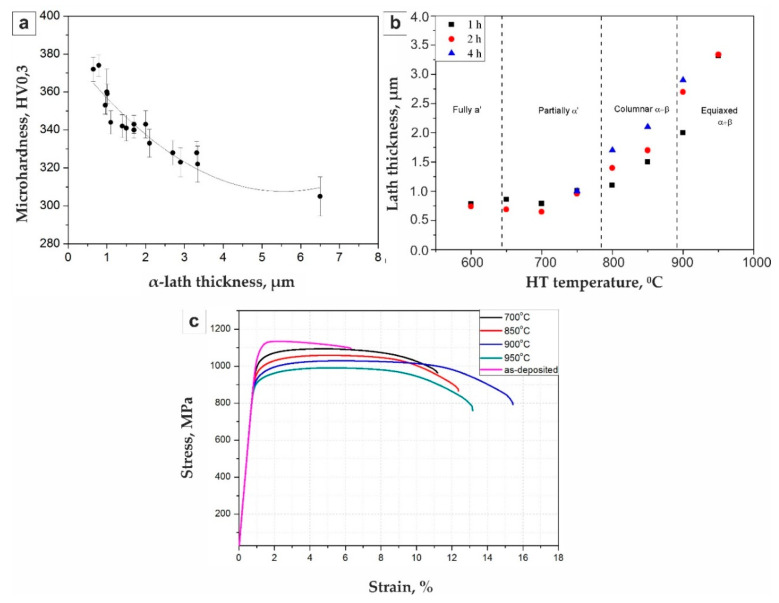
Experimental microhardness measurements for different α-lath thickness values; the tendency line was plotted using polynomial regression analyses (**a**); experimental data of α-lath thickness versus an aging time graph for different temperatures (**b**); typical stress–strain curves of DLD with the Ti-6Al-4V alloy performed with different HT modes (**c**).

The graph of Figure 4a shows the dependence of the plates’ α/α’ thickness on temperature and holding time during heat treatment. As expected, an increase in the temperature and holding time led to an increase in the structure components size for the Ti-6Al-4V alloy. In addition, since, at a holding temperature of 600 °C, the hardness does not change and corresponds to the as-deposition state, the initial phase composition α + α’ + β was probably retained. At temperatures of 700–750 °C, even when the holding time was 4 h, only a partial transition of α’ to equilibrium α + β occurred. Starting from temperatures of 800 °C, a more intense decomposition was observed, and above 850 °C, the growth of the structural components already had a prevailing effect on microhardness. 

Based on the experimental data, the Hall–Petch relationship was plotted for the deposited titanium alloy Ti-6Al-4V using polynomial regression analyses that corresponded to the data presented in [12].

The microhardness gradually decreased with an increase in the α phase plate size (Figure 4a). The microhardness measurement confirmed the decomposition of the metastable α’ phase, beginning at the temperature of 700 °C. A further decrease in hardness with increasing temperature and holding time was associated with an increase of α lamellae size (Figure 4). The Hall–Petch relationship can also be observed for temperature and time variation in Figure 4b. The results of the α lath thickness measurement for all the aging temperatures and times are plotted in Figure 4b. The graph shows that α lath size grew with temperature and time. The lamellae coarsening increased with temperature. The results for various temperatures correlated with the data obtained for the SLM-ed Ti-6Al-4V alloy [26].

**Figure 5:**
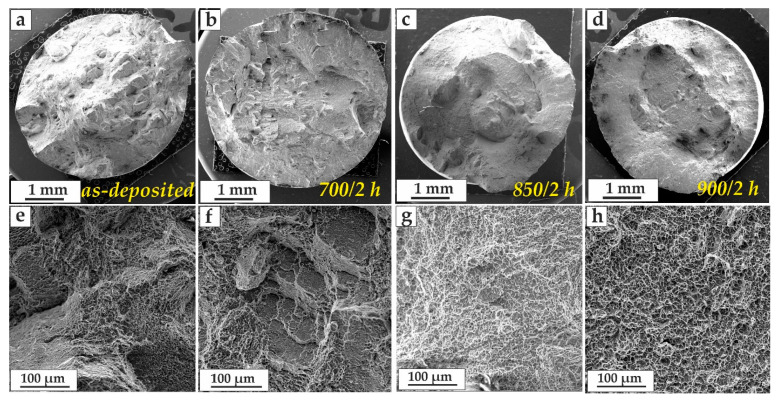
Fracture surface of as-deposited Ti-6Al-4V tensile samples performed in the horizontal direction (*x*-axis) for (**a**,**e**) as-deposited; (**b**,**f**) HT = 700 °C; (**c**,**g**) HT = 850 °C; and (**d**,**h**) HT = 900 °C.

Analysis of the fracture surface showed that an increase in the HT temperature led to a facet size increase, which also corresponded to an increase in ductility and a decrease in tensile strength. Heat treatment at a temperature of 700 °C did not significantly affect the change in the structure; the fracture of this sample was more similar to the fracture of the as-deposited samples, also characterized by inter-crystalline fracture (Figure 5a,b,e,f). The use of heat treatment temperatures above 800 °C led to a partial or complete decomposition of metastable structures. Above 900 °C, the growth of structural components occurred, which also affected the facet size on the fracture surface (Figure 5c,d,g,h).

**Figure 6:**
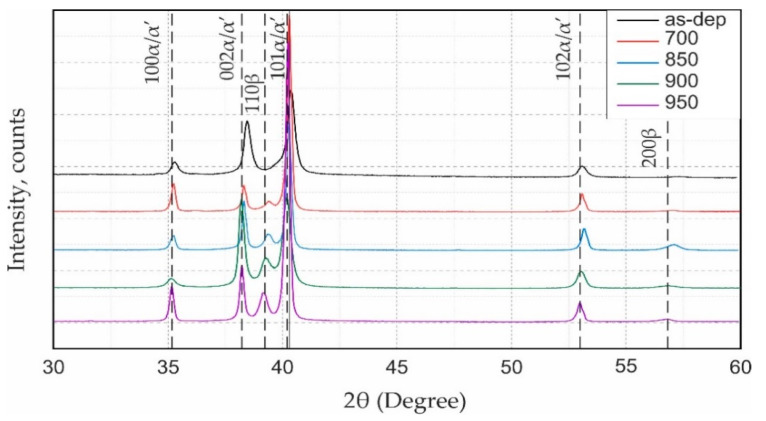
XRD patterns of as-deposited Ti-6Al-4V alloy and after heat treatment.

A comparative analysis of the diffraction patterns of the as-deposited sample and after heat treatment using different temperatures allowed the determination of the features of the change in the intensity phases’ diffraction lines. As-deposited samples had a slight shift of lines that, as noted previously, indicated the formation of a metastable α’ phase due to dissolving more alloying elements in its lattice, which explained the shift [27] (Figure 6). The metastable α’ was partially present in the Ti-6Al-4V up to 900 °C, and diffraction peaks of the α phase had some shifts. Complete α’ phase decomposition was observed above 900 °C. As-deposited XRD-patterns also had peak broadening that indicated internal stresses. After heat treatment, the internal stress level decreased, and the peak shape became narrower. This corresponded to the data presented in the work of T. Ungar [28]. In addition, a change of (101)α and (200)α intensity could be traced. Shunyu Liu et al. associated the intensity of alpha planes variation with decomposition of martensite and the preferred grain orientation changing [29]. Moreover, corresponding with optical micrograps, Figure 4 shows that the growth of structural components is observed with increasing HT temperature. The increasing of alpha plates also influenced peak intensity. 

The diffraction line intensity of the β-phase increase for deposited Ti-6Al-4V samples after heat treatment. In addition, for all heat treatments, the β-phase diffraction lines were broadened. Based on this, it can be concluded that the β-phase had some inhomogeneity in composition and stress level, which was typical for the phase in which decomposition occurred. The position of the β-phase lines was not constant, and indicate a slightly different degree of decomposition depending on the holding temperature. This was especially pronounced on the sample after heat treatment with a holding temperature of 850 °C. This indicated that at a given temperature, the holding time of 2 h was not enough to complete the phase transition processes. 

### 3.2. Heat Treatment of Depositede Ti-6Al-4V with Different Structures

In the deposit samples, depending on thermal cycles, heating and cooling rates, and modes parameters, different types of structures typical for Ti-6Al-4V can be observed. For different types of structures, there can be different ratios of phases: α + β, α + β, and α + α’ + β. The size, distance from the substrate, and the thermal cycle, as previously indicated for various AM technologies of Ti-6Al-4V, had a significant effect on the final structure, phase composition, and the distribution of alloying elements. [30,31]. 

**Figure 7:**
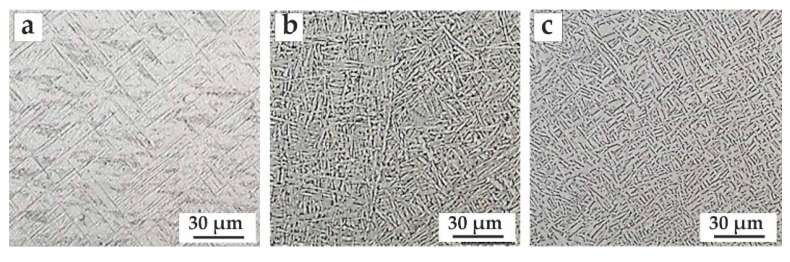
Microstructure of deposited Ti-6Al-4V with different initial structure (**a**) sample 1 (α + α’), (**b**) sample 2(α + β), and (**c**) sample 3(α + α’ + β).

To study the effect of the selected heat treatment mode on three types of initial Ti-6Al-4V structures, samples deposited under different conditions were tested (Table 3). Changes in microstructure and properties depending on conditions have been shown in previous work [32]. It was shown that higher cooling rates were observed at the bottom of the blade near the substrate, which led to the partial or full α’ formation. Above the 40th layer, heat accumulated and an equilibrium structure α + β was formed. In accordance with the above, the selected heat treatment mode was tested on three samples to show good applicability, regardless of the initial structure (Figure 7, Table 3).

Samples with nonuniform structure have different mechanical properties (Table 4). The heat treatment parameters must be selected in such a way that the properties in the initial state do not decrease if they have the required level comparable to the mechanical properties of this alloy in the rolled condition, but, at the same time, so that they can be improved if the required level is not achieved. 

The Table 3 shows that heat treatment led to a similar plasticity level for deposited Ti-6Al-4V samples after HT, although, before there was a significant difference in properties, which was most likely associated with the ratio of equilibrium and non-equilibrium phases in the alloy.

## 4. Conclusions

During a direct laser deposition process from a Ti-6Al-4V powder alloy of aviation parts, which have variable geometric dimensions of the sections due to the thermal cycle’s changes, a non-uniform structure can form that decreases the mechanical properties. As a result, the mechanical properties of the part become heterogeneous, which can lead to premature failure during deposition or decrease working properties. In this paper, the optimal heat treatment mode is selected, which, regardless of the structure, gives a good result and evens out the mechanical properties in the part.

(a) The stabilization of the a’ phase had a strong influence on its decomposition and grain growth during subsequent heat treatment. Lamellae size showed some relation with heating temperature, illustrating the significance of the initial microstructure and heating temperature applied in post-DLD heat treatment. Above the heating temperature 850 °C, α lamella width growth became more intensive (>2.5 µm).

(b) The metastable α’ is partially present in the Ti-6Al-4V up to 900 °C. Complete α’ phase decomposition is observed above 900 °C. XRD results show that the β phase also has some inhomogeneity in composition and stress level that is typical for the phase in which decomposition occurs. Full-phase transformation for DLD-ed Ti-6Al-4V alloy when the temperature of heat treatment is above 900 °C occurs.

(c) An elongation increase an decrease in the tensile strength occurs with a growth in the holding temperature from 700 °C (σ_t_ = 1100 MPa, el = 8.27%) to 900 °C (σ_t_ = 1026 MPa, el = 14.2%). Above 900 °C, a decrease in elongation begins with a simultaneous decrease of tensile strength (σ_t_ = 990 MPa, el = 13% for 950 °C) that is associated with an increase in the α lamellae and beta grain size. The results for all properties are well above ASTM standards for forged (ASTM F1472) and cast Ti6-Al-4V (ASTM F1108).

The best mechanical characteristics of laser-deposited Ti-6Al-4V are ensured by an HT with a temperature of 900 °C and a holding time of 2 h. The selected HT parameters allow the homogeneity of properties and microstructure in DLD-ed Ti-6Al-4V parts with variable thickness and complexity, particularly in deposited GTA blades.

## Figures and Tables

**Table 1:** Technological parameters of the Ti-6Al-4V DLD process.

Power, W	Speed, mm/s	Gas Consumption L/min	Beam Diameter, mm	Powder Consumption, %
1500	30	15	2.8	35–40

**Table 2:** The results of mechanical testing of deposited Ti-6Al-4V cylindrical samples with different HT modes.

HT	Yield Strength, MPa	Tensile Strength, MPa	Elongation, %
as-deposited	1082	1135	5.3
700 °C/AC/2 h	1019	1100	8.27
850 °C/AC/2 h	963	1055	12.2
900 °C/AC/2 h	925	1026	14.2
950 °C/AC/2 h	903.2	990.7	13

**Table 3:** Samples for testing selected heat treatment mode.

Name	Phase Composition	Deposited Blade Part
Sample 1	A + α’	Near substrate
Sample 2	α + β	Until 40th layer
Sample 3	A + α’ + β	After 40th layer

**Table 4:** Mechanical properties of samples deposited with different thermal cycles before and after HT.

№ of Sample	Yield Strength, MPa	Tensile Strength, MPa	Elongation, %	Microhardness HV0.03
sample 1	1082	1135	5.3	385 ± 7.98
sample 1 + HT	888	951	9.7	345 ± 8.61
sample 2	983	1047	10.3	354 ± 5.15
sample 2 + HT	899	960	10,4	339 ± 8.28
sample 3	931	999	5.8	360 ± 6.69
sample 3 + HT	895	958	10.0	341 ± 5.89
